# Structure, Gene Flow, and Recombination among Geographic Populations of a *Russula virescens* Ally from Southwestern China

**DOI:** 10.1371/journal.pone.0073174

**Published:** 2013-09-17

**Authors:** Yang Cao, Ying Zhang, Zefen Yu, Fei Mi, Chunli Liu, Xiaozhao Tang, Yunxian Long, Xiaoxia He, Pengfei Wang, Jianping Xu

**Affiliations:** 1 Laboratory for Conservation and Utilization of Bio-Resources, and Key Laboratory for Microbial Resources of the Ministry of Education, Yunnan University, Kunming, Yunnan, PR China; 2 Department of Biology, McMaster University, Hamilton, Ontario, Canada; University of Sydney, Australia

## Abstract

Mushrooms that are morphologically indistinguishable from *Russula virescens* (Schaeff.) Fr. are among the most popular wild edible mushrooms in Yunnan province, southwestern China. However, almost nothing is known about their biology. This study investigated the diversity and population genetics of a *R. virescens* ally from Yunnan. A total of 210 samples were collected from 13 geographical locations throughout the main distribution range in Yunnan. The patterns of genetic variation within and among these geographic populations were analyzed using sequences from three nuclear and two mitochondrial DNA fragments. Analysis of the ITS sequences revealed that samples from Yunnan showed 3–6% sequence difference from *R. virescens* samples from North America and Europe and formed a distinct clade. Our multilocus population genetic analyses suggested frequent gene flow among geographic populations of the *R. virescens* ally in Yunnan. Interestingly, the nuclear and mitochondrial genes exhibited different levels of gene flow and recombination. We discuss the implications of our results for understanding speciation, reproduction and conservation of this important biological resource.

## Introduction

Ectomycorrhizae (EcM), symbiotic associations between fungi and plants’ roots, are commonly found in natural ecosystems and are indispensable for healthy forests and grasslands. In the complex EcM network, carbohydrates, minerals and water are frequently exchanged between host plants and fungal mycelia [1]–[3] and the nutrients can even be transferred from one tree to another through the EcM networks [4]. An individual tree may have 15 or more different fungal EcM partners at one time [5]. Many EcM fungi can produce fruiting bodies under certain environmental conditions and these fruiting bodies then release basidiospores for dispersal and sexual reproduction. Some of these fruiting bodies are well-known gourmet edible mushrooms such as *Tricholoma matsutake, Cantharellus cibarius, Boletus edulis*, and *Russula virescens*.

Yunnan province in southwestern China is a famous biodiversity hotspot [6]. Its high biodiversity, including that of wild mushrooms, is mainly due to the tremendous variation in environmental and ecological conditions brought about by the complex geographic and climatic factors [7]. Species of *Russula* are among the most important wild edible mushrooms, contributing an estimated thirty percent of the overall edible mushroom trade in Yunnan [8]. Surveys over the past several decades have identified twelve morphological species or species complex in this genus in wild edible mushroom markets in Yunnan [8]. The most common is what the locals call “Qingtoujun” (literarily means the “green-headed” mushroom) and these mushrooms have macro-morphological characteristics indistinguishable from those of *R. virescens* found in Europe and North America. Indeed, most Chinese researchers have traditionally referred to “Qingtoujun” from southwest China as belonging to *R. virescens* or this species complex [9]. Aside from its importance as food for the locals, Qingtoujun has also been used as a Chinese traditional medicine for more than six hundred years [10]. Indeed, the demand for this mushroom both within and outside of China has been increasing since the mid-1990s.

At present, Qingtoujun cannot be artificially cultivated. Like many other wild gourmet mushrooms from this region, the increasing consumer demands can lead to overharvesting. For example, over 70% of the Qingtoujun in wild mushroom markets are small immature fruiting bodies with their caps closed and have relatively little commercial value (personal observation). Furthermore, there are increasing disturbances to the forest ecosystem and loss of habitats associated with this mushroom in this region. At present, the impact of these changes on natural populations of this mushroom remains virtually unknown. Indeed, very little is known about the biology of Qingtoujun, including its ecology, evolution and population genetics. Understanding these fundamental issues could be important for developing sustainable conservation and utilization strategies.

Despite their important ecological roles and economic significance, relatively few studies of the *Russula* genus have been conducted in China [11]. The studies so far have focused on surveys of the species of this genus in China and have primarily used morphological features and borrowed species names originating from European and American literature [11]–[13]. As a result, relatively little is known about the genetic and phylogenetic diversities of this genus in China. Another factor contributing to the limited understanding of *Russula* in China (and elsewhere) has been the dearth of morphological features to reliably distinguish the over 700 published species in this genus. In addition, only a small number of *Russula* species have their DNA sequences represented in databases. The recent application of DNA sequences (including DNA barcodes) for strain and species identifications are beginning to shed light on the taxonomy, systematics, and phylogenetic relationships within this genus. For example, a recent molecular phylogenetic and population genetic study revealed that “Dahongjun” (or the “Big Red Mushroom”) from southern China contained at least three phylogenetically divergent lineages and that none of these lineages belonged to *Russula vinosa*, a morphologically similar species distributed in Europe and North America but has been used to describe Dahongjun in China. Interestingly, the three lineages are largely geographically structured and there is a high level of genetic diversity within each of the lineages in southern China [14].

In this study, we collected and analyzed 210 mushroom specimens from 13 geographical locations in Yunnan. The patterns of genetic variation within and among these geographic populations were analyzed using sequences from five DNA fragments including three from the nuclear genome [the internal transcribed spacer (ITS) region, the second largest subunit of the nuclear RNA polymerase gene (RPB2), the chitin synthase gene (CHSI)], and two from the mitochondrial genome [the ATPase subunit 6 gene (ATP6) and the cytochrome oxidase subunit III gene (COX3)]. The data are used to address the following specific questions. First, how much DNA sequence divergence is there between Qingtoujun from Yunnan and *R. virescens* from Europe and North America? Second, how is genetic variation structured within and among geographic populations of Qingtoujun from Yunnan? Are these populations genetically differentiated as was shown in the “Big Red Mushroom”? And third, are population structures inferred from the nuclear and mitochondrial markers different for geographic populations of Qingtoujun from Yunnan?

## Materials and Methods

### Sampling

**Figure 1:**
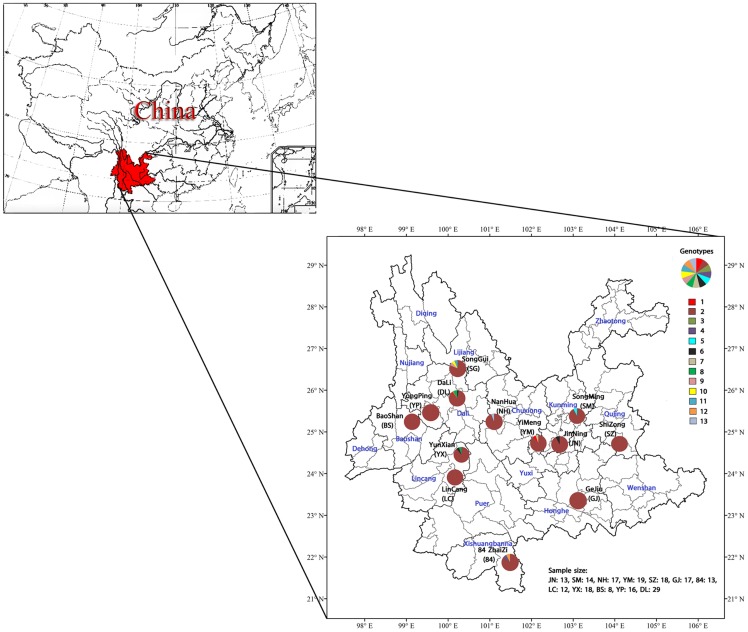
Geographic samples and ITS genotype distributions of the *R. virescens* species complex from Yunnan. Yunnan Province is highlighted in red; the administrative regions within Yunnan province are marked in blue; and the sample collection localities (county/community) are marked in black. Pie charts show the relative frequency of ITS genotypes at each collection locality.

A total of 210 isolates of Qingtoujun were collected from 13 geographical locations in Yunnan province in southwest China during the rainy season (June-August) in 2010 and 2011. Since Qingtoujun is not on the endangered or protected species list, no specific permit is required for picking and trading this mushroom. Our samples were obtained from a combination of sources: local farmers’ markets, roadside mushroom sellers, and our own collection efforts in public lands that required no specific permits to access. Our sampling locations spanned about 600 km from north to south and 500 km from east to west. These 13 local populations belonged to nine Prefectures (an administrative level of government between the county and the provincial governments). The sample size and geographical coordinates for each population are presented in Figure 1.

### DNA Extraction, PCR and Sequencing

Genomic DNAs of all samples were individually extracted using the CTAB method slightly modified for fungi [15]. In this study, five DNA fragments including ITS, RPB2, CHSI, ATP6, and COX3 were analyzed. Relevant sequences obtained from GenBank were used to design primers when necessary. PCR amplifications of these five fragments were conducted using the following primer pairs: ITS4 and ITS5 [16] for the ITS region; ATP6-4S (5′ CAACAATTAATACAATGGTAAG 3′) and ATP6-4A (5′ CCAAAGGAATTAAAGTGAAT 3′) for the mitochondrial ATP6 gene; CHSI-5S (5′ CGATTTCACCGCAAGCAC 3′) and CHSI-5A (5′ CGAACCCTCAGCGTAGTT 3′) for the nuclear CHSI gene; COX3-1S (5′ ACTTTAGGTGCAGTTATG 3′) and COX3-1A (5′ TAGCTCCTCTTCTATCTCCTTG 3′) for the mitochondrial COX3 gene; and RPB2-2S (5′ CAACGGTGTCTGGATGGG 3′) and RPB2-2A (5′ CAGGGAAAGGAATGATACTGG 3′) for the nuclear RPB2 gene. Sequencing was performed by BGI Co. Ltd (Shenzhen, China). Sequences resulting from this study are deposited in Genbank with the following accession numbers: KC598287∼KC598496 (ITS); KC598849∼KC599024 (RPB2); KC599025∼KC599200 (CHSI); KC598497∼KC598672 (ATP6); and KC598673∼KC598848 (COX3).

### Analyses of ITS Sequences

**Figure 2:**
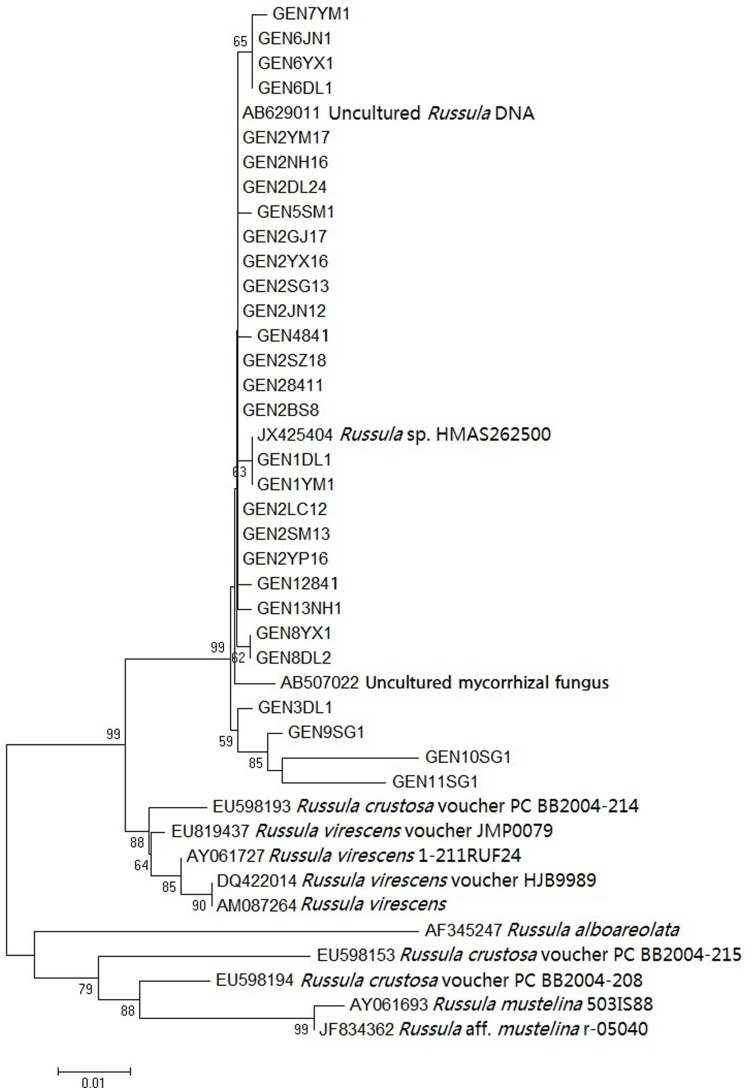
A neighbor-joining tree of the ITS sequences of the 13 genotypes of a *R. virescens* ally from Yunnan and the closely related ITS sequences of genus *Russula* from GenBank. For each ITS genotype (GEN) in our samples, the first number represents the genotype assignment; the characters after represent the county/community from where the strains were sampled; the last number represents the total number of strains belonging to the specific genotype. The reference strains are each represented by their GenBank accession number, the genus abbreviation and the species name. The bootstrap values are obtained from 1000 replicates.

The complete ITS sequences were assembled using DNAStar v. 5.0. These sequences were then used as queries to retrieve closely related sequences with comparable lengths from GenBank (Fig. 2). The retrieved sequences from GenBank as well as our own were aligned using Clustal_X v. 2 [17]. The aligned sequences were visually inspected, adjusted and imported into MEGA 4.0 [18], [19] to identify unique sequence types and to infer the phylogenetic relationships among our strains and between our strains and those from GenBank. A neighbor-joining tree was produced using the Kimura-2-parameter model, and validated using a bootstrap analysis with 1000 repetitions.

### Genetic Diversity and Population Structure

Since the heterozygous sites observed for the ITS gene fragments could be due to its multi-copy nature within individual ribosomal RNA gene clusters and not between homologous chromosomes, our analyses didn’t rely solely on information from the ITS gene. Instead, the population genetic analyses were based on four other DNA fragments that were either single copy nuclear gene (CHSI and RPB2) or mitochondrial genes (ATP6 and COX3). Because fertile mycelia and fruiting bodies in the *Russula* genus analyzed so far have shown genotypes to be consistent with each cell being a diploid and/or dikaryotic, sequences at the two single copy nuclear genes that we obtained directly through PCR from each Qingtoujun fruiting body likely contained information from both alleles.

In order to analyze the two nuclear gene sequence information fully, we first inferred the putative allelic sequences for both the CHSI and the RPB2 loci respectively from each individual mushroom using the Bayesian method by PHASE 2.1 [20]. The inferred haplotype sequences were then used to analyze the relationships among alleles from within the same and different mushroom fruiting bodies using the maximum parsimony algorithm implemented in PAUP*4.0b10 [21]. In addition, the haplotype information was combined into one dataset and imported into GenAlEx6 [22] to calculate the pairwise population *F_ST_* values and determine the potential correlation between genetic and geographical distances (Mantel test). The analysis of molecular variance (AMOVA) was performed to estimate the relative contributions of geographic separation to the overall genetic variation. As mitochondrial genomes are typically homogeneous within individual cells and among cells within a fruiting body [23], sequence variations at the two mitochondrial genes ATP6 and COX3 were analyzed directly as haploid loci. These two mitochondrial genes were analyzed separately from those of the two nuclear genes in order to compare the potential similarities and differences in population structure between these two genomes.

### Relationships among Alleles from Different Loci

The relationships between alleles at these four genes were analyzed using the program Multilocus version 1.3b [24]. Total samples and the clone-corrected samples were used to conduct the following tests. Specifically, two tests were performed for our data, the index of association (*I_A_*) [25] and phylogenetic compatibility. These analyses were performed for the nuclear and mitochondrial genes separately as well as in combination. Since the numbers of SNPs within the mitochondrial genes and nuclear genes were very different (see Results below), the *I_A_* values were adjusted based on the numbers of variable nucleotide sites to obtain the standardized RbarD values [24].

### GenBank Accession Numbers

KC598287 ∼ KC598496 [the internal transcribed spacer (ITS) regions of the nuclear ribosomal RNA gene cluster]; KC598849∼KC599024 [the second largest subunit of the nuclear RNA polymerase gene (RPB2)]; KC599025 ∼ KC599200 [the chitin synthase gene (CHSI)]; KC598497∼KC598672 [ATPase subunit 6 gene (ATP6)]: and KC598673∼KC598848 [the cytochrome oxidase subunit III gene (COX3)].

## Results

### ITS Genotype Distribution and Phylogenetic Analyses

We successfully obtained ITS sequences from all 210 Qingtoujun specimens. Analysis of the 601 aligned nucleotides identified a total of 13 ITS sequence types for the 210 specimens. The distributions of these genotypes among local populations are shown in Fig. 1. The most broadly distributed ITS genotype, genotype 2, was represented by 193 strains and was the most frequently isolated within each of the 13 geographic populations. The second most broadly distributed ITS sequence type was genotype 6, represented in 3 specimens and distributed in three different local populations. Two other ITS genotypes, genotypes 1 and 8 representing two and three specimens respectively, were each found in two different geographic populations. The remaining 9 ITS genotypes were each found in one local population and in one specimen only (Fig. 1).

Phylogenetic analyses of our ITS sequences and those closely related ones from GenBank identified that our samples clustered closely with *R. virescens* from America and Europe (Fig. 2). The DNA sequence similarities between our samples and *R. virescens* were 94∼97%. Our ITS sequence results suggest that our samples were genetically distinct from *R. virescens* samples from other major geographic regions.

### Sequence Variation within the Other Four Sequenced Gene Fragments

Among the 210 specimens, we were able to successfully obtain DNA sequence information from 176 specimens at the four other gene loci. These 176 strains were distributed in all 13 local populations. Below we describe the polymorphisms found in each of the four gene fragments.

#### The mitochondrial ATP6 gene

A total of four variable sites were found among the 337 aligned nucleotide sites for the ATP6 gene. No evidence of heterozygosity was found for any of the sites within any of the 176 samples, consistent with mitochondrial homogeneity within each of the mushroom samples. These four variable sites separated the 176 strains into four sequence types or haplotypes (Fig. S1). Haplotype 1 was the most abundant, represented by more than 90% of the strains and was found in all 13 populations that we sampled. Haplotype 2 was shared by seven strains from five populations while haplotype 3 was shared by nine strains from six populations. Haplotype 4 was found in only one strain from YiMen.

#### The mitochondrial COX3 gene

A total of 296 nucleotides were obtained and analyzed for each of the 176 samples. Two variable sites were identified and these polymorphic sites separated the 176 strains into three haplotypes. The three haplotypes (1, 2, and 3) contained 4, 72 and 100 strains respectively. The four strains in haplotype 1 were distributed in four different populations while haplotypes 2 and 3 were found in every geographic population (Fig. S2).

#### The nuclear CHSI and RPB2 genes

Different from the above two mitochondrial genes, the two nuclear genes sequenced here were found to contain heterozygous nucleotide sites within many of the individual mushroom samples. Because the samples we used were fruiting bodies which, in most basidiomycete mushrooms, represented the diploid or dikaryotic phase of their life cycle, we hypothesized that the heterozygous sites in our strains were most likely due to sequence divergence between the two alleles within each individual at the specific loci. The differences were unlikely to be due to paralogous copies of the genes because many individuals were completely homozygous at either or both the CHSI and the RPB2 loci. Therefore, to analyze the allelic relationships among our samples, we first inferred the allelic sequences within each individual at each of the two loci. This was accomplished using the program PHASE 2.1 [20].

At the CHSI gene fragment, 354 nucleotide sites were aligned and analyzed. A total of 32 variable sites were found among the 176 strains. PHASE analyses inferred a total of 82 haplotypes. The two most frequent haplotypes were haplotype 1 and 13. Haplotype 1 was found 101 times (out of 352 total  = 176 individuals with two alleles per individual at the locus) distributed in 10 of the 13 populations. Haplotype 13 was found 93 times and distributed in all 13 populations. Among the 82 inferred unique haplotypes of the CHSI gene, 58 were found only in one population each. The relationships among the haplotypes are presented in Fig. S3. As shown in Fig. S3, the allelic relationship analyses revealed no clear haplotype clustering based on geography, consistent with frequent gene flow among geographic populations.

At the RPB2 gene fragment, 429 nucleotide sites were aligned. A total of 25 variable sites were found among the 176 strains. PHASE analyses inferred a total of 76 haplotypes at the RPB2 gene fragment. The most frequent haplotype in our sample was haplotype 8, with a total count of 71 and distributed in 12 populations. Thirty-five of the 76 haplotypes were found only in one population each. The relationships among the haplotypes at this locus are presented in Fig. S4. Similar to that observed for the CHSI gene fragment, there was no clear geography-based clustering of haplotypes at this locus.

### Population Structure

The haplotype information obtained above was used to understand the population structure of *R. virescens* from Yunnan province. To identify the overall population structure as well as to compare the potential differences in population structure between the nuclear and mitochondrial genomes, we analyzed three datasets. The first included information from all four loci; the second from only the two nuclear loci; and the third from only the two mitochondrial loci. The results of our analyses are shown in Tables S1, S2 and S3 and are briefly summarized below.

In the analyses including all four genes, the pairwise population *F_ST_* values ranged from 0.012 (between GeJiu and DaLi separated by about 227 kilometers) to 0.123 (between ShiZong and BaoShan which are about 484 kilometers apart) (Table S1). In total, 42 of 78 population pairs showed statistically significant genetic differentiation while the remaining 36 pairs showed no significant difference between each other (Table S1). Though the values were slightly different, the same two pairs of populations showed the lowest (0.011) and highest (0.124) *F_ST_* values for the dataset that included only the two nuclear genes (Table S2). However, in the analyses of the two genes from mitochondria (Table S3), the pair of populations from ShiZong and SongGui separated about 426 kilometers had the lowest FST value (0.002) while that between 84 ZhaiZi and BaoShan separated by about 472 kilometers had the highest value (0.138). Interestingly, of the 42 pairs of populations that showed statistically significant differentiations in nuclear genes, only 7 showed the same level of statistical significance based on the mitochondrial genes. The remaining 71 of the 78 population pairs showed no significant differentiation based on the two mitochondrial genes.

The overall analysis of molecular variance (AMOVA) based on the three types of data revealed that over 90% of the genetic variation was found within local populations (Table 1). While AMOVA identified no evidence of differentiation among populations for the mitochondrial genes with 98% of the genetic variation found within local populations, low but overall significant genetic differentiation was found for the nuclear genes (P<0.05), consistent with the pairwise population differentiation tests.

**Table 1:** Summary results of AMOVA within and among geographic populations of the *R. virescens* species complex from Yunnan, southwestern China.

Source		d.f.	SS	MS	Estimated variance	Percentage	*P*
Four genes combination	Between regions	8	52.908	6.613	0.087	2%	0.090
	Among populations within regions	4	18.986	4.747	0.111	3%	0.100
	Within populations	163	556.492	3.414	3.414	95%	0.010
	Total	175	628.386	14.774	3.612		
Mitochondrial genes	Between regions	8	14.269	1.784	0.029	2%	0.370
	Among populations within regions	4	5.020	1.255	0.000	0%	0.360
	Within populations	163	223.210	1.369	1.369	98%	0.240
	Total	175	242.500	4.408	1.399	100%	
Nuclear genes	Between regions	8	38.638	4.830	0.058	3%	0.030
	Among populations within regions	4	13.966	3.491	0.121	5%	0.020
	Within populations	163	333.282	2.045	2.045	92%	0.010
	Total	175	385.886	10.366	2.223	100%	

d.f., Degrees of freedom; SS, sum of squared observations; MS, mean of squared observations; Est. var., estimated variance; % Var., percentage of total variance; PhiRT, proportion of the total genetic variance between regions; PhiPR, proportion of the total genetic variance among populations within a region; PhiPT, proportion of the total genetic variance among individuals within populations.

To determine whether the observed genetic differentiation between pairs of *R. virescens* populations from Yunnan was correlated to their geographical distances, we conducted a Mantel test. As shown in Fig. S5, S6, S7, the *F_ST_* values of four genes combination (Fig. S5), genes from only the nuclei (Fig. S6), and genes from only the mitochondria (Fig. S7) all exhibited slightly positive correlation to geographical distances. However, none of these correlations were statistically significant.

### Evidence for Recombination

Nucleotide variations within and among the four genes were used to examine the associations among alleles at the four loci. In this analysis, each variable nucleotide site was treated as a locus and different nucleotides at the same site were viewed as different alleles. For the total samples, the observed RbarD values were 0.0539, 0.1418 and 0.0679 for the four genes, the two mitochondrial genes only, and the two nuclear genes only datasets respectively (Table 2). None of these values were significantly different from the randomized recombining datasets (P = 0.792, 0.96, 0.668 respectively). In the second test, the phylogenetic incompatibility test, the results for the four genes and the two nuclear genes only both failed to reject the null hypothesis of random recombination (P = 0.551, 0.410 respectively). Although the mitochondrial genes rejected the null hypothesis of random recombination, clear evidences of phylogenetic incompatibility were found between these two genes (Table 3). Our results are thus consistent with limited but unambiguous recombination within the mitochondrial genomes of *R. virescens* populations in Yunnan.

**Table 2:** Results of multilocus linkage disequilibrium analyses for the *R. virescens* species complex from Yunnan, southwestern China.

Population	*I_A_* (*P* value)	RbarD (*P* value)	Phylogenetic compatibility (*P* value)
Four genes (n = 176)	2.9917 (0.792)	0.0539 (0.792)	0.4327 (0.551)
Four genes (clone-corrected, n = 158)	2.7323(0.944)	0.0488(0.944)	0.4326(0.512)
Mitochondrial genes (n = 176)	0.6691 (0.960)	0.1484 (0.960)	0.8000 (0.041)
Mitochondrial genes (clone-corrected, n = 7)	0.1942(0.912)	0.0389(0.912)	0.8000 (1.000)
Nuclear genes (n = 176)	3.3953 (0.668)	0.0679 (0.668)	0.4642 (0.410)
Nuclear genes (clone-corrected, n = 152)	3.0621 (0.852)	0.0605 (0.852)	0.4642 (0.458)

**Table 3:** Evidence for phylogenetic incompatibility between the two mitochondrial loci in samples of the *R. virescens* species complex from Yunnan, southwest China.

	COX3 site 2 (C)	COX3 site 2 (T)
**ATP6 site 1 (A)**	70	97
**ATP6 site 1 (T)**	2	7
**ATP6 site 2 (A)**	2	6
**ATP6 site 2 (T)**	70	98
**ATP6 site 3 (A)**	70	98
**ATP6 site 3 (G)**	2	6

Our analyses of clone-corrected samples showed results similar to those of the total samples. The only difference was for that between the two mitochondrial genes. Specifically, in the clone-corrected analyses, both the Index of Association and the phylogenetic compatibility tests showed results consistent with random recombination in the mitochondrial genome.

## Discussion

### ITS Sequence Variation and Cryptic Speciation

The internal transcribed spacer (ITS) regions within the nuclear ribosomal RNA gene cluster have been broadly used to identify fungal species and are the recommended DNA barcode for fungi [26]. Recent results showed that the ITS regions have the highest resolving power for discriminating closely related species with a high PCR and sequencing success rate across a broad range of fungi [26]. Furthermore, there are more fungal ITS sequences in GenBank than any other DNA fragments. However, when analyzing population genetic structure, the ITS sequences should be used with caution since ITS is multi-copied within the nuclear ribosomal gene cluster. As a result, whatever variation we observe within individual organisms could be due to variation among repeats on the same chromosome and/or variation between sequences on homologous chromosomes, making it difficult to formulate the data for analyses [14].

Significant divergence in ITS sequences has been found between geographically distinct populations in a variety of fungi, including several basidiomycete mushrooms [14], [27]–[31]. In this study, based on the neighbor-joining tree of ITS, our strains of Qingtoujun clustered most closely with the strains of *R. virescens*, consistent with their morphological similarity. However, our samples showed 3∼6% ITS sequence divergence with *R. virescens* samples from Europe and America. The sequences from GenBank (AB629011, JX425404, AB507022) that showed >99% sequence identities to our sequences were all from unidentified samples from Japan or China. The significant ITS sequence divergence between ours and those from Europe and North America suggest that our samples likely belong to a novel species different from but closely related to the European and American *R. virescens*, similar to those found in many other fungi [27]–[31]. However, information from more gene loci for the European and American specimens as well as from more detailed ecological and morphological comparisons are needed in order to define our samples as belonging to a new species. In the absence of such information, we prefer to be conservative and call our samples as a *R. virescens* ally, belonging to the same *R. virescens* species complex as those from Europe and North America.

### Gene Flow

Though evidence for statistically significant population differentiation was found, our study revealed strong evidence of gene flow among populations of the *R. virescens* species complex in Yunnan. First, we found broad ITS genotype sharing among geographic populations, with the most frequent ITS genotype shared by 193 strains from all 13 local populations. Second and more importantly, our analyses based on data from the four gene fragments through several tests (haplotype inferences, *F_ST_* value calculation, Mantel tests, and AMOVA) all showed consistent evidences for gene flow. Specifically, the haplotype inferences identified that three of the loci (CHSI, ATP6, and COX3) each had at least one haplotype distributed in all 13 local populations analyzed here. The fourth loci RPB2 had one haplotype (haplotype 8) that was found in 12 of the 13 local populations. Indeed, AMOVA results showed that over 90% of the observed genetic variation were found within individual local populations. The Mantel test also showed no statistically significant correlation between the *F_ST_* values and geographical distances, suggesting that the observed gene flow is frequent and unlikely to be impeded by the hundreds of kilometers between populations examined here. There are many valleys and mountains in Yunnan and several are located in our sampled regions. While these geographic barriers may temporally facilitate divergence, they did not seem to have prevented gene flow among these populations.

At present, large-scale population genetic studies of ectomycorrhizal mushrooms are still relatively limited. However, previous studies have revealed examples of both frequent gene flow among geographic populations for some species and significant differentiation for others. For example, Roy et al. [32] found that several French populations of *Laccaria amethystina* separated by over 450 km apart showed limited genetic differentiation with the *F_ST_* values showing no correlation with geographic distance, similar to what we found here. The ectomycorrhizal mushroom *Tricholoma matsutake* from southwestern China also showed limited (though statistically significant) genetic differentiation. The limited genetic differentiation between geographic populations in *R. virescens* could be due to several factors including natural long-distance dispersal of basidiospores [33] and human-aided dispersal through mushroom trading that could mix spores from distant populations. Unlike in the matsutake mushroom where fruiting bodies just before their caps open command significantly higher prices than those with open caps, there is relatively limited price difference between immature and mature Qingtoujun. As a result, mature fruiting bodies of Qingtuojun are commonly found in mushroom markets. The trading of mature Qingtoujun fruiting bodies could bring spores from one area to another and enhance gene flow among geographic populations. However, the pickers we spoke to all traded Qingtoujun within 30 km radius of where they had been picked. So for this particular species, movement of fruiting bodies is less likely to be a factor in enhancing gene flow over longer distances than direct spore dispersals in nature.

### Mode of Reproduction

Associations among alleles from within the same or different loci are commonly used as indicators for sexual (i.e. recombination) or asexual reproduction in natural populations of microorganisms. Testing allelic associations is typically accomplished through the analyses of multilocus linkage disequilibrium [29], [34]. In our study, we used the nucleotide variations from four DNA fragments to infer the relative roles of sexual and asexual reproduction in natural populations of *R. virescens*. Frequent sexual reproduction would generate random associations among alleles while extended asexual reproduction would generate significant non-random associations among alleles. Our analyses showed that alleles between the two nuclear genes as well as between the nuclear and mitochondrial genes were associated with each other not significantly different from random, consistent with prevalent sexual reproduction in natural populations of *R. virescens* species complex in Yunnan. Our results thus emphasize the importance of maintaining mature fruiting bodies in natural environments to conserve the genetic diversity and ensure future reproduction of this mushroom.

### Differences in Patterns of Genetic Variation Between Mitochondrial and Nuclear Genes

Our analyses revealed several differences between the two mitochondrial and two nuclear genes in our samples of *R. virescens* species complex from Yunnan. First, more polymorphic sites and higher numbers of haplotypes were observed for nuclear genes than for mitochondrial genes. For example, four and three haplotypes were observed for the mitochondrial ATP6 and COX3 genes respectively. In contrast, 82 and 76 haplotypes were inferred from the nuclear CHSI and RPB2 genes respectively. This large difference was mainly due to the limited number of polymorphic nucleotide sites for the two mitochondrial genes: 6 polymorphic sites total for the combined 633 nucleotides of the ATP6 and COX3 gene fragments vs. 57 polymorphic sites for the 783 bp of the CHSI and RPB2 genes. Our results contrast with those found in human fungal pathogens *Candida albicans* [35] and *Cryptococcus gattii* [36] that showed significantly higher sequence divergence for mitochondrial genes than in nuclear genes. At present, the reasons for the difference between our samples and the two human fungal pathogens remain unknown. In animals including humans, there is a much higher level of polymorphism for mitochondrial genes than for nuclear genes.

The second difference between mitochondrial and nuclear genes in our samples was in the patterns of allelic association and linkage equilibrium. As expected, the nuclear genes showed random associations among their alleles while alleles at the two mitochondrial genes showed significant non-random associations. However, limited but clear evidence of phylogenetic incompatibility was found between the two mitochondrial genes, consistent with low level of mitochondrial recombination. Our results add to the growing literature on mitochondrial recombination in natural populations of fungi [23], [36].

Aside from the levels of polymorphism, geographic distributions of the observed genetic variations also showed differences between mitochondrial and nuclear genes. Specifically, evidence of genetic differentiation was very limited among populations for mitochondrial genes while statistically significant genetic differentiation was observed for nuclear genes. Such a pattern suggests differential gene flow between the nuclear and mitochondrial genes with the mitochondrial genome showing a greater rate of gene flow than nuclear genes among populations of the *R. virescens* species complex in Yunnan. At present, the mechanism for the observed difference in gene flow between the two genomes is not known. However, one possibility is that after basidiospores disperse to new environments and germinate to form homokaryons, existing resident dikaryons instead of self-sterile homokaryons might be involved in fertilizing the immigrant homokaryon. As has been shown in laboratory experiments, this type of mating would result in a new dikaryon with a recombinant nuclear genome containing the mitochondria of the immigrant [23]. Since basidiospores are likely the dominant agents for dispersal and genetic exchange among populations, such matings would create a more uniform mitochondrial genotype distribution across geographic populations than those of nuclear genes. Another possibility is differential natural selection that favors the specific mitochondrial types throughout the region while maintains a certain level of geographic-specific nuclear elements. These two hypotheses are not mutually exclusive and analyses of long-term samples might help us identify the cause(s) for these differences.

In conclusion, our study is the first to examine the population biology of the *R. virescens* species complex from southwest China. This mushroom is one of the most economically and ecologically important in that region. Our analyses identified that the Yunnan populations of this species had ITS sequences distinctly different from those from Europe and the Americas. Abundant genetic variations were identified within the Yunnan samples at the sequenced loci, especially at the two nuclear gene loci. We found limited geographic structuring, frequent gene flows, and little evidence for significant correlation between geographic and genetic distances among these populations. In addition, our analyses revealed differences in the patterns of nuclear and mitochondrial genetic variation. The mechanisms for such differences and the potential speciation of the Yunnan populations of the *R. virescens* species complex from those in other regions require further analyses.

## Supporting Information

Figure S1 **A maximum parsimony tree of the mitochondrial ATP6 sequences of the** ***R. virescens*** **species complex in Yunnan, southwest China.** A total of four ATP6 haplotypes were found in our samples. For each ATP6 haplotype (HAP), the first number represents the haplotype assignment; the characters after represent the county/community from where the strains were sampled; the last number represents the total number of strains belonging to the specific haplotype. Only representative sequences of unique haplotypes from each location are shown.
(TIF)

Figure S2 **A maximum parsimony tree of the mitochondrial COX3 sequences of the** ***R. virescens*** **species complex in Yunnan, southwest China.** A total of three COX3 haplotypes were found in our samples. For each COX3 haplotype (HAP), the first number represents the haplotype assignment; the characters after represent the county/community from where the strains were sampled; the last number represents the total number of strains belonging to the specific haplotype. Only representative sequences of unique haplotypes from each location are shown.
(TIF)

Figure S3 **A maximum parsimony tree of the nuclear CHSI gene sequences of the** ***R. virescens*** **species complex in Yunnan, southwest China.** A total of 82 unique CHSI haplotypes were found in our samples. For each CHSI haplotype (HAP), the first number represents the haplotype assignment; the characters after represent the county/community from where the strains were sampled; the last number represents the total number of strains belonging to the specific haplotype. Only representative sequences of unique haplotypes from each location are shown.
(TIF)

Figure S4 **A maximum parsimony tree of the nuclear RPB2 gene sequences of the** ***R. virescens*** **species complex in Yunnan, southwest China.** A total of 76 unique RPB2 haplotypes were found in our samples. For each RPB2 haplotype (HAP), the first number represents the haplotype assignment; the characters after represent the county/community from where the strains were sampled; the last number represents the total number of strains belonging to the specific haplotype. Only representative sequences of unique haplotypes from each location are shown.
(TIF)

Figure S5 **Relationship between the levels of genetic differentiation (** ***F_ST_*** **values) estimated based on all four gene fragments (CHSI, RPB2, ATP6 and COX3) and geographical distances between pairs of local populations of the** ***R. virescens*** **species complex from Yunnan, southwestern China.** The X-axis represents geographic distances (in kilometers) between pairs of local populations and the Y-axis represents *F_ST_* values between pairs of local populations.
(PDF)

Figure S6 **Relationship between the levels of genetic differentiation (** ***F_ST_*** **values) estimated based on the two nuclear genes (CHSI and RPB2) and geographical distances between pairs of local populations of the** ***R. virescens*** **species complex from Yunnan, southwestern China.** The X-axis represents geographic distances (in kilometers) between pairs of local populations and the Y-axis represents *F_ST_* values between pairs of local populations.
(PDF)

Figure S7 **Relationship between the levels of genetic differentiation (** ***F_ST_*** **values) estimated based on the two mitochondrial genes (ATP6 and COX3) and geographical distances between pairs of local populations of the** ***R. virescens*** **species complex from Yunnan, southwestern China.** The X-axis represents geographic distances (in kilometers) between pairs of local populations and the Y-axis represents *F_ST_* values between pairs of local populations.
(PDF)

Table S1 **Genetic differentiation (** ***F_ST_*** **values) estimated based on information from all four gene fragments (CHSI, RPB2, ATP6, and COX3) between all pairs of geographic populations of the** ***R. virescens*** **species complex in Yunnan, southwest China.**
(DOCX)

Table S2 **Genetic differentiation (** ***F_ST_*** **values) estimated based on information from the two nuclear gene fragments (CHSI and RPB2) between all pairs of geographic populations of the** ***R. virescens*** **species complex in Yunnan, southwest China.**
(DOCX)

Table S3 **Genetic differentiation (** ***F_ST_*** **values) estimated based on information from the two mitochondrial gene fragments (ATP6 and COX3) between all pairs of geographic populations of the** ***R. virescens*** **species complex in Yunnan, southwest China.**
(DOCX)
