# Supplementary figures and images for: Structure, Gene Flow, and Recombination among Geographic Populations of a Russula virescens Ally from Southwestern China
Source: PLoS One. 2013 Sep 17;8(9):e73174. doi: 10.1371/journal.pone.0073174 (PMC3775738; doi:10.1371/journal.pone.0073174)

Fig. S5

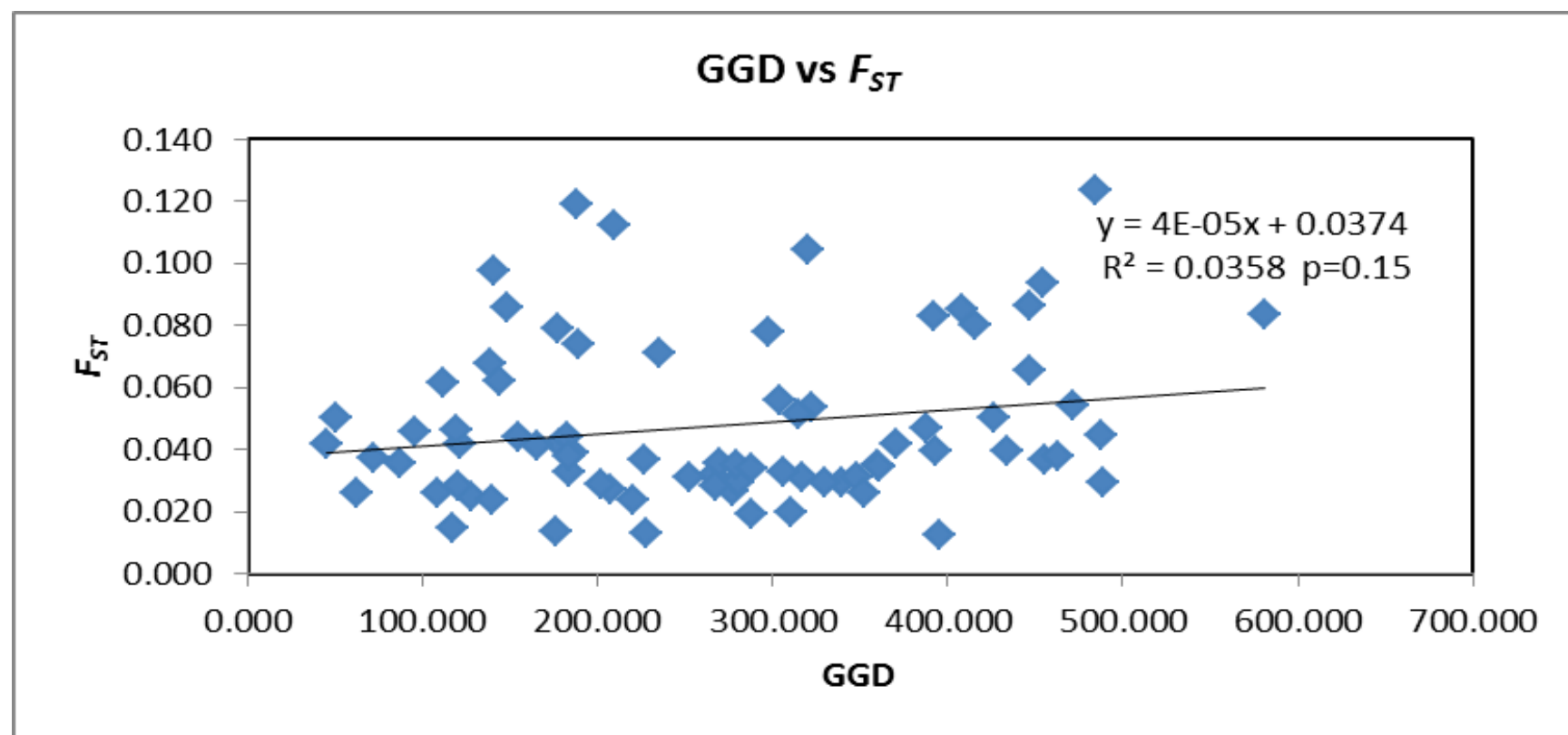

Supplement: Figure S5 — Relationship between the levels of genetic differentiation ( FST values) estimated based on all four gene fragments (CHSI, RPB2, ATP6 and COX3) and geographical distances between pairs of local populations of the R. virescens species complex from Yunnan, southwestern China. The X-axis represents geographic distances (in kilometers) between pairs of local populations and the Y-axis represents FST values between pairs of local populations. (PDF) [file pone.0073174.s005.pdf]

Fig. S6

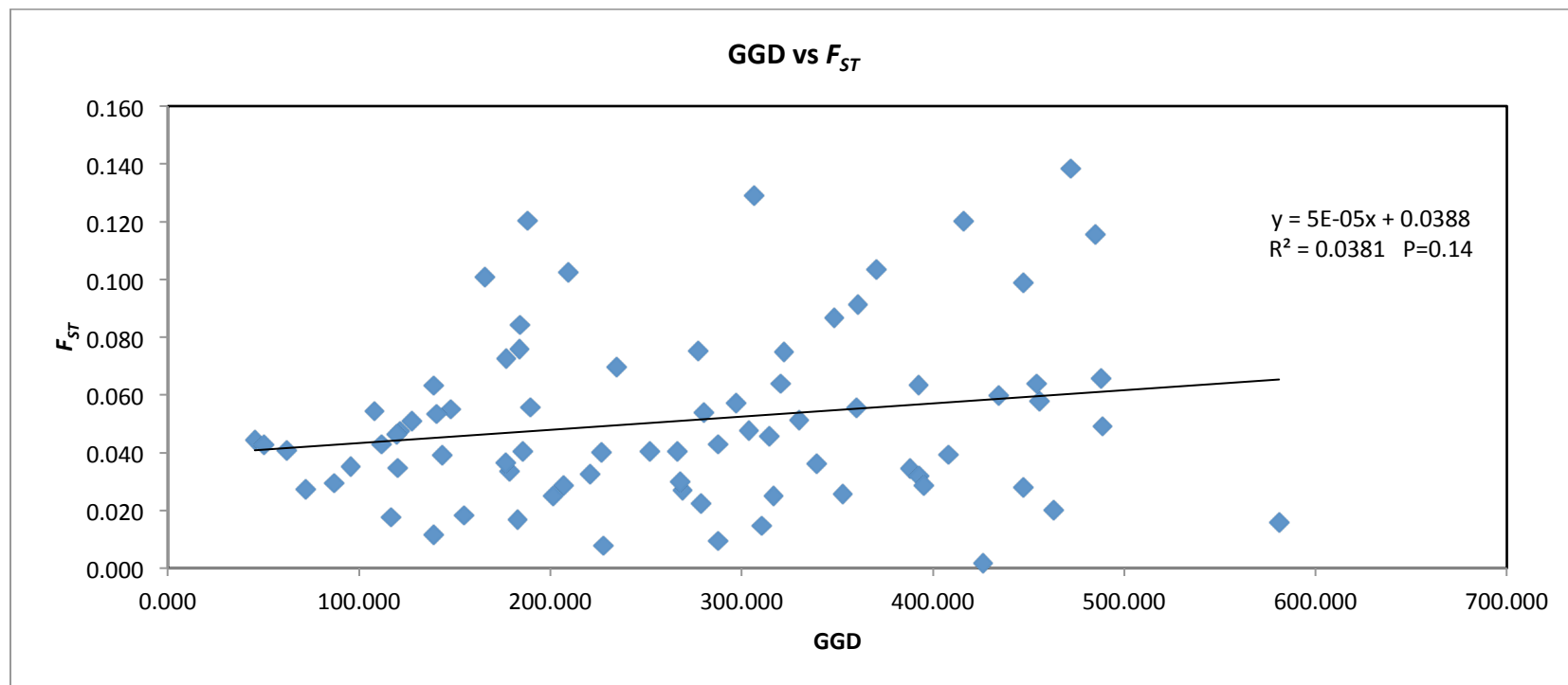

Supplement: Figure S6 — Relationship between the levels of genetic differentiation ( FST values) estimated based on the two nuclear genes (CHSI and RPB2) and geographical distances between pairs of local populations of the R. virescens species complex from Yunnan, southwestern China. The X-axis represents geographic distances (in kilometers) between pairs of local populations and the Y-axis represents FST values between pairs of local populations. (PDF) [file pone.0073174.s006.pdf]

Fig. S7

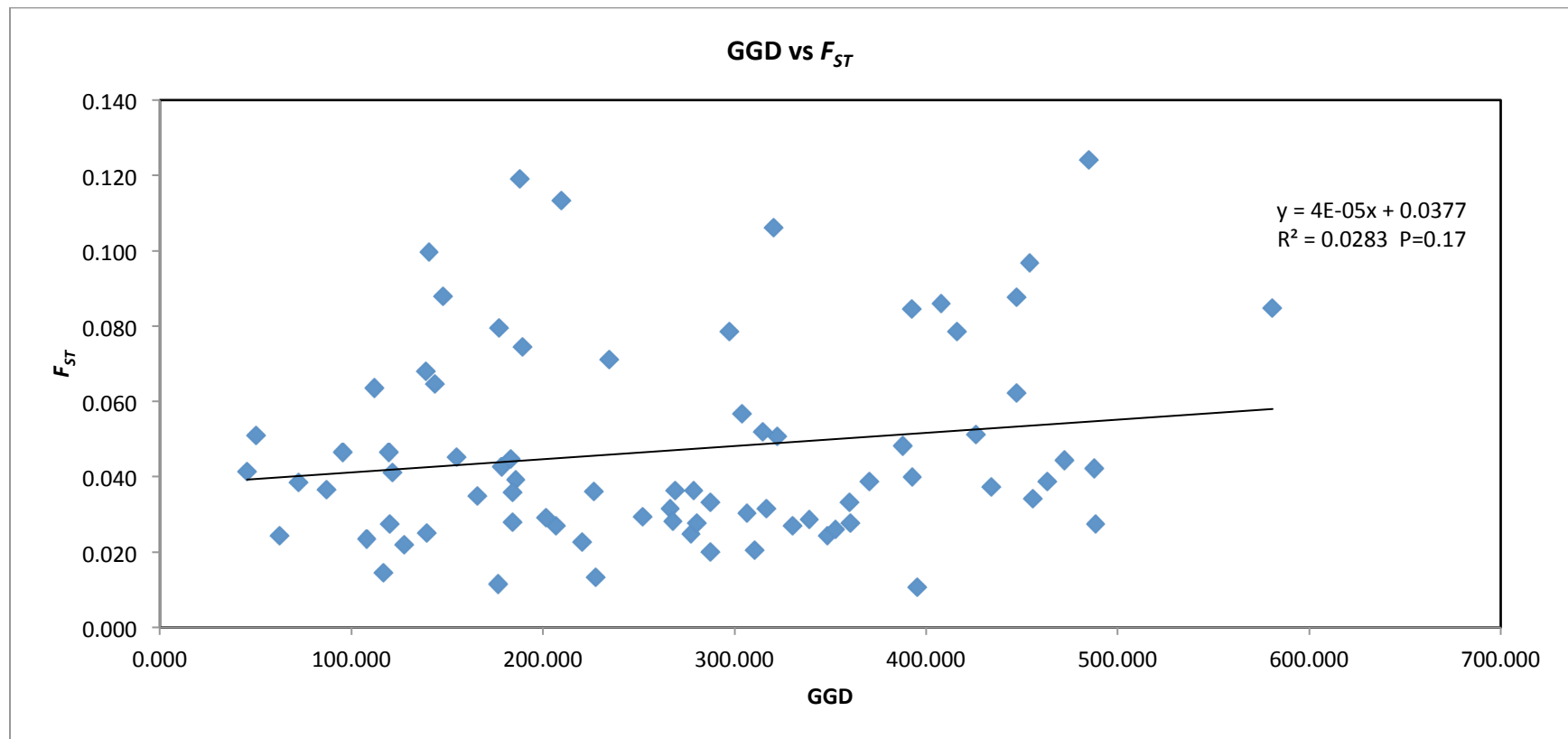

Supplement: Figure S7 — Relationship between the levels of genetic differentiation ( FST values) estimated based on the two mitochondrial genes (ATP6 and COX3) and geographical distances between pairs of local populations of the R. virescens species complex from Yunnan, southwestern China. The X-axis represents geographic distances (in kilometers) between pairs of local populations and the Y-axis represents FST values between pairs of local populations. (PDF) [file pone.0073174.s007.pdf]
