# Supplementary material for: Structure, Gene Flow, and Recombination among Geographic Populations of a Russula virescens Ally from Southwestern China
Source: PLoS One. 2013 Sep 17;8(9):e73174. doi: 10.1371/journal.pone.0073174 (PMC3775738; doi:10.1371/journal.pone.0073174)
Supplement: Table S2 — Genetic differentiation ( FST values) estimated based on information from the two nuclear gene fragments (CHSI and RPB2) between all pairs of geographic populations of the R. virescens species complex in Yunnan, southwest China. (DOCX) [file pone.0073174.s009.docx]

Table S2. Genetic differentiation (*F_ST_*) estimated based on the two nuclear genes between all pairs of geographic populations of the *R. virescens* species complex in Yunnan, southwestern China

| JN_Km | SM_Km | NH_Cx | YM_Yx | SZ_Qj | GJ_Hh | 84_Xs | LC_Lc | Yx_Lc | BS_Bs | YP_Yp | DL_Dl |  |
| --- | --- | --- | --- | --- | --- | --- | --- | --- | --- | --- | --- | --- |
| 0.037 |  |  |  |  |  |  |  |  |  |  |  | SM_Km |
| 0.088^**^ | 0.043^**^ |  |  |  |  |  |  |  |  |  |  | NH_Cx |
| 0.041^*^ | 0.015 | 0.024 |  |  |  |  |  |  |  |  |  | YM_Yx |
| 0.100^**^ | 0.064^**^ | 0.025 | 0.039^**^ |  |  |  |  |  |  |  |  | SZ_Qj |
| 0.045^*^ | 0.023 | 0.028^*^ | 0.012 | 0.045^**^ |  |  |  |  |  |  |  | GJ_Hh |
| 0.039 | 0.034 | 0.079^**^ | 0.033^*^ | 0.088^**^ | 0.031^*^ |  |  |  |  |  |  | 84_Xs |
| 0.036 | 0.029 | 0.074^**^ | 0.036 | 0.086^**^ | 0.031 | 0.030 |  |  |  |  |  | LC_Lc |
| 0.030 | 0.020 | 0.065^**^ | 0.027 | 0.085^**^ | 0.027 | 0.028 | 0.024 |  |  |  |  | YX_Lc |
| 0.024 | 0.048^*^ | 0.113^**^ | 0.057^**^ | 0.124^**^ | 0.062^**^ | 0.044 | 0.035 | 0.041 |  |  |  | BS_Bs |
| 0.051^*^ | 0.026 | 0.080^**^ | 0.036 | 0.097^**^ | 0.037^*^ | 0.042 | 0.028 | 0.022 | 0.051^*^ |  |  | YP_Dl |
| 0.028 | 0.020 | 0.028^**^ | 0.013 | 0.040^**^ | 0.011 | 0.027^*^ | 0.029 | 0.025^*^ | 0.047^**^ | 0.038^**^ |  | DL_Dl |
| 0.106^**^ | 0.052^*^ | 0.036 | 0.033 | 0.051^*^ | 0.039 | 0.085^**^ | 0.079^**^ | 0.071^**^ | 0.119^**^ | 0.068^**^ | 0.046^*^ | SG_Dl |

*,0.01 ≤ *P* ≤ 0.05; **, *P* ≤ 0.01
