# Supplementary material for: Structure, Gene Flow, and Recombination among Geographic Populations of a Russula virescens Ally from Southwestern China
Source: PLoS One. 2013 Sep 17;8(9):e73174. doi: 10.1371/journal.pone.0073174 (PMC3775738; doi:10.1371/journal.pone.0073174)
Supplement: Table S3 — Genetic differentiation ( FST values) estimated based on information from the two mitochondrial gene fragments (ATP6 and COX3) between all pairs of geographic populations of the R. virescens species complex in Yunnan, southwest China. (DOCX) [file pone.0073174.s010.docx]

Table S3. Genetic differentiation (*F_ST_*) estimated based on two mitochondrial genes between all pairs of geographic populations of the *R. virescens* species complex from Yunnan, southwest China

| JN_Km | SM_Km | NH_Cx | YM_Yx | SZ_Qj | GJ_Hh | 84_Xs | LC_Lc | Yx_Lc | BS_Bs | YP_Yp | DL_Dl |  |
| --- | --- | --- | --- | --- | --- | --- | --- | --- | --- | --- | --- | --- |
| 0.029 |  |  |  |  |  |  |  |  |  |  |  | SM_Km |
| 0.055 | 0.034 |  |  |  |  |  |  |  |  |  |  | NH_Cx |
| 0.044 | 0.018 | 0.054 |  |  |  |  |  |  |  |  |  | YM_Yx |
| 0.053 | 0.043 | 0.075 | 0.040 |  |  |  |  |  |  |  |  | SZ_Qj |
| 0.018 | 0.033 | 0.054 | 0.037 | 0.017 |  |  |  |  |  |  |  | GJ_Hh |
| 0.103^*^ | 0.058^*^ | 0.120^**^ | 0.055 | 0.028 | 0.040 |  |  |  |  |  |  | 84_Xs |
| 0.027 | 0.036 | 0.056 | 0.040 | 0.039 | 0.025 | 0.129^*^ |  |  |  |  |  | LC_Lc |
| 0.041 | 0.015 | 0.039 | 0.029 | 0.063^**^ | 0.051^*^ | 0.091^**^ | 0.041 |  |  |  |  | YX_Lc |
| 0.087 | 0.034 | 0.102^*^ | 0.048 | 0.115^**^ | 0.099^*^ | 0.138^**^ | 0.101 | 0.048 |  |  |  | BS_Bs |
| 0.075 | 0.026 | 0.073 | 0.022 | 0.064 | 0.060 | 0.066 | 0.076 | 0.051^*^ | 0.043 |  |  | YP_Dl |
| 0.030 | 0.009 | 0.035 | 0.008 | 0.032 | 0.029 | 0.049^*^ | 0.025 | 0.012 | 0.046 | 0.027 |  | DL_Dl |
| 0.064 | 0.046 | 0.084 | 0.043 | 0.002 | 0.020 | 0.016 | 0.057 | 0.070^*^ | 0.120 | 0.063 | 0.035 | SG_Dl |

*,0.01 ≤ *P* ≤ 0.05; **, *P* ≤ 0.01
